# Editorial: Tumor markers of respiratory tumors: from bench to bedside

**DOI:** 10.3389/fmed.2025.1551629

**Published:** 2025-01-14

**Authors:** Weiguo Huang, Chengsheng Yang, J. Luis Espinoza, Lin Wu, Jianpin Wen

**Affiliations:** ^1^Guangxi Key Laboratory of Tumor Immunity and Microenvironment Regulation, Guilin Medical University, Guilin, China; ^2^School of Health Sciences, College of Medical Pharmaceutical and Health Sciences, Kanazawa University, Kanazawa, Japan; ^3^Hunan Cancer Hospital, Xiangya School of Medicine, Central South University, Changsha, China; ^4^Hunan Medical College General Hospital, Huaihua, China

**Keywords:** tumor markers, lung cancer, nasopharyngeal cancer, cancer risk factors, prognostic assessment

Malignant tumors of the respiratory system are mainly comprised of lung cancer and nasopharyngeal cancer (NPC). The incidence rate and mortality of lung cancer is highest among all malignant tumors; it is also one of the most harmful. NPC, meanwhile, has the highest incidence rate in Southeast and East Asia.

Due to the lack of obvious symptoms, early lung cancer is difficult to diagnose, and its diagnostic methods mainly include conventional methods, such as chest X-rays and Computerized tomography (CT) scans, as well as early biomarker detection.

In this topic, a study was conducted based on existing databases to explore the relationship between the biomarker angiotensin-converting enzyme 2 (ACE2) and the incidence of lung cancer. Chen et al. summarized the relationship between ACE2 and the risk of lung cancer. The relevant SNPs were then taken from the lung cancer GWAS dataset and two-sample Mendelian randomization (MR) was used to ascertain if ACE2 is causally linked to the risk of developing lung cancer. This research revealed a significant causal link between ACE2 and the risk of getting lung cancer. These findings may have implications for public health measures aimed at reducing the incidence of lung cancer.

Meanwhile, new lung cancer biomarkers are constantly being discovered; Xia et al. found a new lung cancer marker—Triggered transposable element derivative 1 (TIGD1)—through bioinformatics methods and revealed TIGD1's potential as a biomarker for diagnosing and predicting lung cancer. Cellular studies confirmed TIGD1's involvement in cancer cell proliferation, invasion, and migration. Integrating computational analysis with empirical studies will enhance understanding of TIGD1's significance in NSCLC and open avenues for further research into targeted therapies.

Due to its insidious onset, early diagnosis of NPC is difficult in clinical practice. CT and MRI play important roles in the diagnosis of early NPC. In this topic, Lei et al. used meta-analysis to evaluate the comparative diagnostic accuracy of [18F]FDG PET/CT vs. [18F]FDG PET/MRI in identifying lymph node metastases in individuals with NPC. They found that [18F]FDG PET demonstrates high sensitivity and specificity in identifying lymph node metastasis in NPC. Furthermore, [18F]FDG PET/CT exhibits comparable sensitivity and specificity to [18F]FDG PET/MRI. The occurrence of metastasis in NPC is one of the main reasons for its poor prognosis, and it is of great significance to accurately determine the impact of metastasis on the prognosis of NPC. Xiao et al. established a nomogram model of lung metastasis of NPC as a supplement to TNM staging. They found that age, T-stage, radiation, chemotherapy, and brain metastases can affect the OS in NPC with lung metastasis. The nomogram model also offers better clinical net benefits than TNM staging. Xiao et al successfully established a nomogram model of NPC lung metastasis that can be used as a supplement to TNM staging and provide reference for clinicians.

We still need to search for more sensitive and newer diagnostic biomarkers, while also investigating new methods, technologies, and means for early diagnosis and prognosis judgment of respiratory malignancies using clinical big data. As shown in this Research Topic, “*Tumor Markers of Respiratory Tumors: from Bench to Bedside*,” the early diagnosis and prognosis assessment of malignant tumors in the respiratory system are of great significance.

